# Short-term outcomes of enhanced recovery after surgery protocol in robotic-assisted McKeown esophagectomy for esophageal cancer: a single-center retrospective cohort study

**DOI:** 10.3389/fonc.2023.1150945

**Published:** 2023-12-12

**Authors:** Xia Xu, Jiajun Xiong, Zhijie Xu, Zhi Hu, Guha Alai, Lulu Yu, Shaofeng Xia, Yidan Lin

**Affiliations:** ^1^ Department of Pathology, Affiliated Jinhua Hospital, Zhejiang University School of Medicine, Jinhua, China; ^2^ Department of Thoracic Surgery, Jiujiang First People’s Hospital, Jiujiang, China; ^3^ Department of Thoracic Surgery, West China Hospital of Sichuan University, Chengdu, China; ^4^ Health Management Center, Jiujiang First People’s Hospital, Jiujiang, China

**Keywords:** enhanced recovery after surgery, esophageal cancer (ec), robotic-assisted McKeown esophagectomy, perioperative management, outcomes

## Abstract

**Background:**

This study aimed to evaluate the short-term outcomes of enhanced recovery after surgery (ERAS) protocol in perioperative robotic-assisted McKeown esophagectomy (RAME) among esophageal cancer patients.

**Methods:**

For this retrospective study, all patients who had undergone RAME with esophageal cancer using ERAS protocol and conventional management strategy at the surgery center of our hospital from February 2019 to March 2022 were performed for analysis.

**Results:**

A total of 211 patients were included. Compared to the conventional group, the ERAS group has shorter median operative time [207 (147.5-267.5) *vs*. 244 (183-305), P<0.001], time to first flatus (P<0.001), time to out-of-bed activity (P=0.045), and time to liquid diet (P<0.001). In addition, the ERAS group has lower postoperative pain scores (3.62 ± 0.87 *vs*. 4.54 ± 0.91), shorter duration of analgesia pump [2 (1-3) *vs*. 3 (2.5-5.5)], shorter postoperative hospital stay [(9 (6-47) *vs*. 11 (6-79)], shorter postoperative hospital stay within neoadjuvant treated patients [8 (7-43) *vs*. 13 (8-67], shorter postoperative ICU stay [1 (0-7) *vs*. 2 (0-15)], and less reoperation rate (7.6% *vs*. 16.8%). Furthermore, the overall complication rate was significantly lower in the ERAS group (26.1%) than in the conventional group (50.4%). Notably, the ERAS group had lower thoracic fluid drainage volume than the conventional group on postoperative 2-7 days (P<0.05).

**Conclusions:**

The application of ERAS protocol in esophageal cancer patients treated with RAME showed advantages of quick postoperative recovery in contrast to the conventional management strategy.

## 1 Introduction

As the sixth leading cause of cancer-related death worldwide, esophageal cancer accounts for approximately 300,000 deaths each year (1), with an increasing incidence in recent years that seriously threatens life and health of patients (2, 3). For a growing proportion of patients with esophageal cancer, comprehensive treatments based on radical surgery with or without neoadjuvant or adjuvant therapy are still the most important (4). Compared with traditional open surgery, minimally invasive esophagectomy (MIE) has been proven to be safe and feasible, which can reduce postoperative complications (especially pulmonary-related complications), accelerate postoperative recovery, shorten hospital stay, as well as achieve satisfied short-term efficacy (5).

With the development of MIE year by year, some problems are gradually revealed in terms of its long learning curve, difficult anastomosis, and the higher incidence of anastomotic leakage and reoperation. The advent of da Vinci robot has provided a new treatment option for esophageal cancer. Since Horgan et al. (6) first reported using robot-assisted minimally invasive esophagectomy (RAMIE) in 2003, the number of RAMIE has also grown year over year. Over the next decade, increasing evidence has reported the development of RAMIE and confirmed its safety, feasibility, and good short-term efficacy in terms of operation time, blood loss, lymphadenectomy, perioperative complications, and mortality (7, 8). Among them, robotic-assisted McKeown esophagectomy (RAME) is one of the most frequently used surgical methods in esophageal cancer and has advantages in lymphadenectomy, especially upper mediastinal and cervical lymphadenectomy.

Although RAME has the advantages of minimally invasive, mild pain, and concealed incision to meet the requirements of cosmetology (9), there still exist problems among most patients with esophageal cancer, such as decreased cellular immune function, malnutrition, poor cardiopulmonary function (10), frequent complications, prolonged hospital stays, and high hospitalization costs (11). Therefore, perioperative treatment and care are of great importance with the purpose of improving therapeutic effects, reducing complications, and alleviating pain. In recent years, enhanced recovery after surgery (ERAS) protocol has provided a new platform and mode for patients undergoing surgical treatment for the sake of maximizing improvement of both physical and psychological trauma, as well as maximizing reduction of perioperative stress reactions and complications (12). ERAS protocol has been reported primarily on colorectal cancer surgery but rarely in esophageal cancer. This study aimed to investigate the short-term efficacy of ERAS strategy in perioperative RAME among esophageal cancer patients.

## 2 Patients and methods

### 2.1 Patients

The institutional review board approved this retrospective study, and the study conformed to the Declaration of Helsinki and Strengthening the Reporting of Cohort Studies In Surgery 2019 (STROCSS 2019) (13), and all participants signed informed consent. Patients with esophageal cancer undertaking resection by RAME (da Vinci Xi Surgical System, Intuitive Surgical Inc., Sunnyvale, CA) were included from February 2019 to March 2022. The inclusion criteria were as follows: (1) aged 20 to 80 years; (2) first detected and endoscopically confirmed esophageal cancer; (3) preoperative evaluation showed no distant metastases and suitable for RAME; (4) preoperative clinical stage of I to III. Patients were excluded if (1) they had tumors located at the cervical esophagus or gastro-esophageal junction; (2) they had a history of thoracic or abdominal surgery; (3) they were IV to VI in the American Society of Anesthesiologists (ASA) physical status classification system; (4) they had other malignancies; (5) they had missing clinical data. To prevent surgeon bias, all participating surgeons had experienced more than 40 RAME cases per year and completed a learning curve before the study (14). Finally, all eligible patients were divided into the ERAS group (n=92) and the conventional group (n=119) based on different perioperative management strategies.

### 2.2 Surgical technique

RAME was independently performed for both groups at the surgery center in our hospital. Right bronchial closure was performed, with moderate use of capnothorax to aid atelectasis, adequately expose the surgical field and blow smoke from electrocautery device manipulation. Surgical procedures included freeing the esophagus and lymphadenectomy through the right chest, freeing the stomach and regional abdominal lymphadenectomy through the upper abdomen, and tubular anastomosis of the gastroesophagus through the left neck.

Intrathoracic operation: The patient was placed in the left 90° decubitus position with the robotic arm entered from the dorsal direction. A 3-arm 4-puncture method was used: a robotic endoscope (12mm Trocar) was placed in the 6th intercostal space of the right posterior thoracic axillary line, robotic 1-arm and 2-arm (8mm Trocar) were placed in the 3rd intercostal space of the right midthoracic axillary line and the 9th intercostal space of the subscapular angle line, respectively. And the 5th or 7th intercostal space of the midaxillary line was used as an auxiliary operation hole (12mm Trocar). Artificial pneumothorax was established with a pressure 6-8 mmHg. The mediastinal pleura was opened along the esophagus, the azygos arch was transected, and the esophagus was freed upper to thoracic inlet and the lower to esophageal opening of diaphragm. Regional lymphadenectomy was carefully conducted at the left/right recurrent laryngeal nerve, paraesophageal, and subcarinal. After the intrathoracic operation, a silicone drainage tube was placed from the apical chest through the mediastinal esophageal bed, and the tube was connected to a water-sealed bottle.

Abdominal operation: The patient was placed in the reverse trendelenburg position, and the robotic arm entered from the head side direction. A 3-arm 5-puncture method was used: a robotic endoscope (12mm Trocar) was placed 2cm beside the left umbilicus, and robotic 1-arm and 2-arm (8mm Trocar) were placed 1cm below the costal margin of the left anterior axillary line and 6cm above the umbilicus of the right midclavicular line, respectively. And an auxiliary operation hole (5mm and 12mm) was placed 2cm beside the right umbilicus and the right anterior axillary line, respectively. Artificial pneumoperitoneum was established with a pressure 12-15 mmHg. An ultrasound knife was used to open the gastrocolic ligament along greater curvature to reserve the right gastroepiploic vascular arch. Lesser omentum was opened, left gastric vessels were dissected, ligated and cut off, and regional lymphadenectomy was performed. The proximal stomach and abdominal esophagus were freed, pericardial lymphadenectomy was performed, and diaphragmatic hiatus was opened to communicate with thoracic cavity. A midline epigastric incision of about 4cm was performed to create a tubular stomach. An oblique incision at the anterior border of the sternocleidomastoid muscle at the left neck was performed to expose and free the cervical esophagus, and the tubular stomach was pulled from the abdominal cavity along the esophageal bed to the neck for esophagogastric anastomosis.

### 2.3 Perioperative management

The ERAS group had a shorter fasting period before surgery and did not require bowel preparation. In contrast, the conventional group received routine intraoperative anesthesia and treatment. In the ERAS group, an endotracheal tube was removed in the operating room immediately after surgery to limit fluid infusion, enteral nutrition was started early after surgery, liquid diet was resumed as early as possible, and patients were encouraged to exercise as soon as possible. In addition, the chest drain was removed when the drainage was less than 100 mL/day. On the other hand, the conventional group received traditional postoperative treatment. The details of the perioperative strategies in the two groups were listed in **Table 1**.

**Table 1:** Perioperative strategies in two groups.

Management protocols	ERAS group	Conventional group
Preoperative	a. Smoking cessation 2w before surgery. ERAS protocol, purpose, significance, and cooperation were explained. b. Food fasting and liquid fasting 6h and 2h before surgery, respectively. A small intake of carbohydrates (200mL) 4 h before surgery. c. No bowel preparation and preanesthetic medication. d. Preoperative pain guidance to relieve the tension of patients and their families. e. Active cardiopulmonary exercise (Based on routine pulmonary function exercises such as blowing up balloons and climbing stairs, load abdominal breathing exercise was added, and effective cough training was encouraged).	a. Routine oral education (To enable patients to be educated about the surgery and to be able to cooperate with the therapeutic practice) b. Fasting 8 h before surgery c. Routine bowel preparation (Cleansing enema the night before surgery and gastric tube placed the morning of the operation)
Intraoperative	a. Inhaled anesthesia (sevoflurane), and remifentanil and propofol-based intravenous anesthesia were used as anesthesia maintenance methods for surgery. b. Maintained body temperature including pre-emptive skin warming, operating room temperature maintaining at 25°C, blanket applying, and fluid warming. c. Controlled fluid intake to reduce fluid retention and tissue edema. d. Use of antibiotics 30min before surgery.	a. General anesthesia (Intravenous anesthesia with remifentanil and propofol) b. Temperature in operating room c. Conventional infusion (Maintained blood pressure) d. No use of antibiotics before surgery
Postoperative	a. Thoracic epidural analgesia pump (Sufentanil) combined with intravenous drip of nonsteroidal anti-inflammatory drugs. Distracted the patient from the pain and sensitivity by playing light music, video, etc. b. Parenteral and enteral nutrition were administered daily on day 1 and day 2 after surgery. Liquid diet was encouraged to start on day 3 after surgery, while stopping parenteral nutrition and reducing enteral nutrition. c. Upper gastrointestinal imaging was performed on day 5 after surgery, and oral intake was continued if there was no anastomotic fistula. d. Immediate out-of-bed activity in general ward after leaving the postoperative recovery room and permission from doctors and nurses. e. Removal of the urinary catheter 24h after surgery. f. Strengthened recovery exercise. g. Removal of chest drains when the output was 100 mL/day.	a. Patient-controlled intravenous analgesia combined with oral opioids b. Water ingestion 12h after surgery, and liquid diet 2-5d after first flatus c. Upper gastrointestinal radiography was performed on day 7 after surgery to ascertain anastomotic integrity d. Out-of-bed activity was encouraged early e. Removal of the urinary catheter 72h after surgery f. Removal of chest drains when the output was 50 mL/day

### 2.4 Monitoring indexes and definitions

Baseline and clinicopathological parameters were compared between ERAS and conventional groups, including age, gender, body mass index (BMI), smoking and drinking history, preoperative complications, tumor location and type, neoadjuvant treatment regimen, and clinical TNM stage. The primary endpoints of this study were first flatus time, time to out-of-bed activity, time to liquid diet, postoperative pain score, duration of analgesic pump, postoperative hospital stay or that received neoadjuvant therapy, ICU length of stay, reoperation rate, in-hospital mortality or 30-day mortality postoperatively, and incidence of various postoperative complications. The secondary endpoints were postoperative chest drainage volume, preoperative anesthesia time, operation time, blood loss, conversion rate, radicality of surgery, and related pathological outcomes.

In terms of tumor localization, tumors that were usually 20-25, 25-30, and 30-40 cm away from the incisors under endoscopy were defined as upper-, mid-, and lower-thoracic esophageal cancer, respectively. Postoperative pain index was scored using the numeric pain rating scale (NPRS) to assess the patient’s pain on the first day after surgery (15). Clinical and pathological stages of tumor were evaluated according to the TNM definition by the 8th edition of the American Joint Committee on Cancer (AJCC) (16). R0 resection was defined as > 1mm from all resection margins, R1 resection was defined as microscopic residual tumor, and R2 resection was defined as macroscopic residual tumor (17). Postoperative complications including pulmonary complications, cardiac complications, wound infection, and bleeding were determined according to Clavien-Dindo classification (18). Anastomotic leakage, vocal cord paralysis, and chylothorax were defined according to the Esophagectomy Complications Consensus Group (ECCG) (19).

### 2.5 Statistical analysis

Numerical differences between two groups were assessed by chi-square test or Fisher’s exact test for categorical variables, and Mann–Whitney U test or Student’s t-test for continuous variables. The threshold for significance was P=0.05. All statistical analyses were conducted using GraphPad prism software, Version 9.1.1 (GraphPad Prism Software Inc., San Diego, CA).

## 3 Results

### 3.1 Baseline characteristics of patients

A total of 211 patients with esophageal cancer undertaking resection by RAME were included in the final analysis. There were 92 cases in the ERAS group with an average age of 61.5 ± 8.5 years, and 119 cases in the conventional group with an average age of 62.7 ± 8.3 years. Demographics and tumor characteristics of patients in two groups were shown in **Table 2**. No significant differences were found in gender (P=0.304), age (P=0.531), and BMI (P=0.162) between the two groups. ERAS group had more smoking (80.4% *vs*. 79.8%, P=0.913) and drinking history (65.2% *vs*. 63.9%, P=0.839), while the conventional group had more preoperative complications (37.0% *vs*. 33.7%, P=0.622). Most tumors (89.57%) were located at the mid- and lower-thoracic esophagus, and squamous cell carcinoma (83.89%) was more commonly diagnosed. 21.7% and 21.8% of patients in the ERAS and conventional groups received neoadjuvant therapy, respectively. In addition, patients in the conventional group showed more advanced clinical TNM stages (clinical stage II: 63.0% *vs*. 53.3%, clinical stage III: 15.2% *vs*. 14.1%).

**Table 2:** Baseline characteristics in two groups.

Variables	ERAS group (n=92)	Conventional group (n=119)	P value
Age (years), mean ± SD	61.5 ± 8.5	62.7 ± 8.3	0.304
Gender, n (%)			
Male	78 (84.8)	97 (81.5)	0.531
Female	14 (15.2)	22 (18.5)	
BMI (kg/m^2^), n (%)	23.1 ± 3.3	23.7 ± 2.9	0.162
Smoking, n (%)	74 (80.4)	95 (79.8)	0.913
Alcohol consumption, n (%)	60 (65.2)	76 (63.9)	0.839
Comorbidity, n (%)			
Yes	31 (33.7)	44 (37.0)	0.622
No	61 (66.3)	75 (63.0)	
Tumor location, n (%)			
Upper-thoracic	10 (10.9)	12 (10.1)	0.918
Mid-thoracic	43 (46.7)	59 (49.6)	
Lower-thoracic	39 (42.4)	48 (40.3)	
Type of malignancy, n (%)			
Squamous cell carcinoma	81 (88.0)	96 (80.7)	0.149
Adenocarcinoma	11 (12.0)	23 (19.3)	
Neoadjuvant therapy, n (%)			
Chemoradiotherapy	13 (14.1)	16 (13.4)	0.986
Chemotherapy	1 (1.1)	1 (0.8)	
Radiotherapy	6 (6.5)	9 (7.6)	
Clinical TNM stage, n (%)			
I	30 (32.6)	26 (21.8)	0.208
II	49 (53.3)	75 (63.0)	
III	13 (14.1)	18 (15.2)	

### 3.2 Intraoperative and pathological outcomes

**Table 3** displayed surgical and pathological outcomes in the two groups. The mean preoperative anesthesia time was 23.7 ± 4.1 and 23.2 ± 3.9 minutes in the ERAS and conventional groups, respectively (P=0.368). Median operative time was significantly shorter in the ERAS group than in the conventional group [207 (147.5-267.5) *vs*. 244 (183-305), P<0.001]. However, the two groups had no significant difference in intraoperative blood loss (P=0.489). There were 6 (6.5%) and 9 (7.6%) cases converting to open surgery in the ERAS group and conventional group, separately, mainly due to extensive tissue adhesions, intraoperative bleeding or circulatory instability. In addition, neither the R0 resection rate (94.6% *vs*. 90.8%) nor the median number of lymph node harvest [24 (16-32) *vs*. 23 (14-31)] was statistically different between the two groups (P=0.485 and P=0.563).

**Table 3:** Intraoperative and pathological outcomes in two groups.

Variables	ERAS group (n=92)	Conventional group (n=119)	P value
Preoperative anesthetic time (min), mean ± SD	23.7 ± 4.1	23.2 ± 3.9	0.368
Operative time (min), median (IQR)	207 (147.5-267.5)	244 (183-305)	<0.001
Blood loss (ml), median (IQR)	200 (100-400)	200 (100-500)	0.489
Conversions, n (%)	6 (6.5)	9 (7.6)	0.771
Radicality of surgery, n (%)			
R0	87 (94.6)/	108 (90.8)	0.485
R1	4 (4.3)	7 (5.9)	
R2	1 (1.1)	4 (3.3)	
Lymph nodes harvest, median (IQR)	24 (16-32)	23 (14-31)	0.563
Tumor size (cm), median (IQR)	3.5 (1.5-5.5)	4 (2-6)	0.738
Tumor differentiation, n (%)			
Well	24 (26.1)	32 (26.9)	0.960
Moderate	42 (45.7)	52 (43.7)	
Poor	26 (28.3)	35 (29.4)	
Pathological TNM stage, n (%)			
0/I	21 (22.8)	35 (29.4)	0.182
II	39 (42.4)	56 (47.1)	
III	32 (34.8)	28 (23.5)	

Regarding the pathological outcomes of the two groups, the median tumor length was 3.5cm (1.5-5.5) and 4cm (2-6) in the ERAS and conventional groups, respectively, with a P value greater than 0.05 (P=0.738). And there were 24 (26.1%) and 32 (26.9%) cases with well-differentiated tumors in the ERAS group and conventional group, respectively, with a P value greater than 0.05 (P=0.960). Compared with the conventional group, the ERAS group had a higher TNM stage, but there was no statistical difference (P=0.182). Specifically, the ERAS and conventional groups comprised 22.8% (21/92) and 29.4% (35/119) of patients with tumor *in situ* or stage I, 42.4% (39/92) and 47.1% (56/119) of patients with stage II, and 34.8% (32/92) and 23.5% (28/119) of patients with stage III tumors, respectively.

### 3.3 Postoperative outcomes

The short-term clinical results were presented in **Table 4**. The ERAS group had shorter time to first flatus (P<0.001), time to out-of-bed activity (P=0.045), and time to liquid diet (P<0.001). Postoperative pain scores (ERAS *vs*. conventional: 3.62 ± 0.87 *vs*. 4.54 ± 0.91) and duration of analgesia pump [ERAS *vs*. conventional: 2 (1-3) *vs*. 3 (2.5-5.5)] were significantly different between the two groups (P<0.001). Postoperative hospital stay in the ERAS group [(9 (6-47)] was significantly shorter than that in the conventional group [11 (6-79)], with a P value 0.018. Also, the ERAS group had significantly shorter ICU length of stay [1 (0-7)] than the conventional group [2 (0-15)], with a P value less than 0.001. Of note, the patients who received neoadjuvant therapy in the ERAS group [8 (7-43)] had less postoperative hospital stay than that in the conventional group [13 (8-67), (P<0.001)]. The reoperation rate in the ERAS group (7.6%) was also remarkably shorter than that in the conventional group (16.8%), with a P value 0.047. In addition, one patient (1.1%) died during hospitalization in the ERAS group, and three (2.5%) died in the conventional group, mainly due to pulmonary infection or hemorrhagic shock. All the above patients died within 30 days after surgery.

**Table 4:** Postoperative outcomes in two groups.

Variables	ERAS group (n=92)	Conventional group (n=119)	P value
First flatus (d), mean ± SD	1.43 ± 0.41	1.98 ± 0.39	<0.001
Time to out-of-bed activity (h), median (IQR)	2 (1.5-4)	3 (2-6)	0.045
Time to oral feeding (d), median (IQR)	3 (1-5)	4 (2-7)	<0.001
Pain score after operation, mean ± SD	3.62 ± 0.87	4.54 ± 0.91	<0.001
Duration of analgesic pump (d), median (IQR)	2 (1-3)	3 (2.5-5.5)	<0.001
Postoperative hospital stay (d), median (IQR)	9 (6-47)	11 (6-79)	0.018
Postoperative ICU stay (d), median (IQR)	1 (0-7)	2 (0-15)	<0.001
Postoperative hospital stay in neoadjuvant treated patients (d), median (IQR)	8 (7-43)	13 (8-67)	<0.001
Reoperation, n (%)	7 (7.6)	20 (16.8)	0.047
30-day mortality postoperatively, n (%)	1 (1.1)	3 (2.5)	0.634
In-hospital mortality, n (%)	1 (1.1)	3 (2.5)	0.634
Complications, n (%)	24 (26.1)	60 (50.4)	<0.001
Pulmonary complications	5 (5.4)	18 (15.1)	0.025
Cardiac complications	3 (3.3)	7 (5.9)	0.519
Anastomotic leakage	5 (5.4)	9 (7.6)	0.590
Bleeding	3 (3.3)	5 (4.2)	1.000
Chylothorax	0 (0)	2 (1.7)	0.506
Vocal cord paralysis	2 (2.2)	3 (2.5)	1.000
Wound infection	5 (5.4)	11(9.2)	0.433
DGE	1 (1.1)	5 (4.2)	0.235

The overall complication rate was significantly lower in the ERAS group (26.1%) than in the conventional group (50.4%) (P<0.001). Specifically, complications in the two groups were pulmonary complications (5.4% *vs*. 15.1%, P=0.025), cardiovascular complications (3.3% *vs*. 5.9%), anastomotic leakage (5.4% *vs*. 7.6%), postoperative bleeding (3.3% *vs*. 4.2%), chylothorax (0% *vs*. 1.7%), vocal cord paralysis (2.2% *vs*. 2.5%), incisional infection (5.4% *vs*. 9.2%), and delayed gastric emptying (DGE) (1.1% *vs*. 4.2%).

### 3.4 Postoperative thoracic fluid drainage

**Figure 1:**
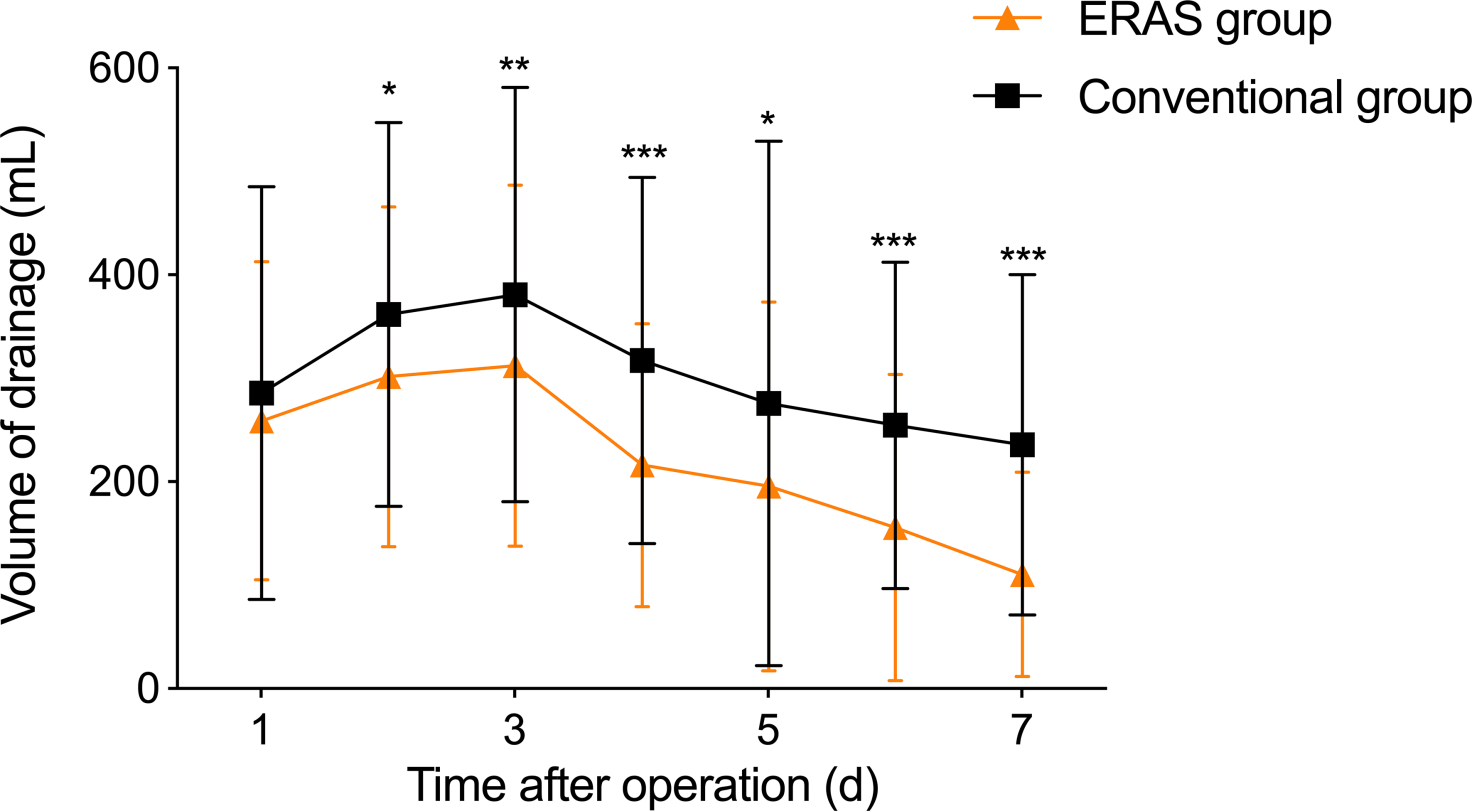
Postoperative thoracic fluid drainage in two groups, ^*^P<0.05, ^**^P<0.01, ^***^P<0.001.

In this study, we continuously monitored postoperative thoracic fluid drainage volume for a week in both groups. As shown in **Table 5**, the ERAS group had significantly lower thoracic fluid drainage volume than the conventional group on postoperative 2-7 days (2^nd^ day: P=0.015, 3^rd^ day: P=0.009, 4^th^ day: P<0.001, 5^th^ day: P=0.011, 6^th^ day: P<0.001, 7^th^ day: P<0.001). Notably, thoracic fluid drainage volume in both groups gradually decreased after peaking on postoperative day 3 (**Figure 1**).

**Table 5:** Postoperative drainage in two groups.

Postoperative thoracic fluid drainage volume	ERAS group (n=92) mean ± SD	Conventional group (n=119) mean ± SD	P value
POD 1 (ml)	258.9 ± 153.7	285.6 ± 199.6	0.289
POD 2 (ml)	301.5 ± 164.3	361.7 ± 185.4	0.015
POD 3 (ml)	312.3 ± 174.6	380.9 ± 200.1	0.009
POD 4 (ml)	216.1 ± 136.7	317.4 ± 176.9	<0.001
POD 5 (ml)	195.6 ± 178.2	275.8 ± 253.6	0.011
POD 6 (ml)	155.7 ± 147.8	254.5 ± 157.9	<0.001
POD 7 (ml)	110.3 ± 98.7	235.8 ± 164.6	<0.001

## 4 Discussion

The concept of ERAS protocol was first introduced by Henrik Kehlet in 1997 in colorectal surgery (20). Over the years, it has evolved into a multidisciplinary approach consisting of surgeons, anesthesiologists, intensivists, physiotherapists, dietitians, and nurses, who will participate in perioperative care of patients and integrate evidence-based management into clinical practice. This multimodal approach has been proven to shorten hospital stays, reduce surgical stress response and morbidity, and speed up recovery (12). Subsequently, the ERAS Society was founded in 2010 and issued guidelines for colorectal, bariatric surgery, gastrectomy, liver surgery, and gynecologic oncology. The implementation of the ERAS concept reduces the cost of overall treatment without compromising the results (21). However, ERAS has low popularity in the treatment of thoracic diseases, especially esophageal cancer, and has not been widely promoted because of the controversy of relevant research (22). In addition, many surgery centers in China do not have a deep understanding of the diagnosis and treatment process or treatment system of esophageal cancer, which in turn implements ERAS progress slowly (23). Therefore, the ERAS concept developed in this study emphasized multidisciplinary collaboration among anesthesiology, pain, and surgery to extend new techniques and concepts to the perioperative management of patients before, during, and after surgery in order to reduce stress response to surgery, maintain nutritional status, as well as promote immune and gastrointestinal function recovery.

Shortening the preoperative fasting time is believed to relieve preoperative tension and anxiety, avoid related organ damage and reduce the length of hospital stay (24). In this study, patients were required food fasting 6h before surgery and liquid fasting 2h before surgery, and no aspiration cough symptoms due to anesthesia during surgery were found. Limited management of fluid replacement aims to reduce the risk of postoperative cardiopulmonary complications, as surgery is an invasive procedure, the operation time is an important indicator affecting the patient’s postoperative recovery. Prolonged surgery stimulates the release of inflammatory cytokines and increases the chance of inflammatory reactions. At the same time, physical damage to organs around the lesion can lead to more severe organ function damage and edema, which aggravate infection, increase the amount of pleural effusion and patient rehabilitation time. This study suggested that compared with the conventional group, the ERAS group had shorter operation time [207 (147.5-267.5) *vs*. 244 (183-305), P<0.001], and less amount of pleural effusion at 2-7 days after operation (P < 0.05). One possible explanation may be attributed to the different preoperative management strategies. In the conventional group, patients undergoing RAME require mechanical enemas the day before surgery, which may cause dehydration and electrolyte imbalance, especially in elderly patients; in the ERAS group, the ERAS protocol eliminated preoperative bowel preparation, then greatly reduced patients’ stress and improved intraoperative safety, which may contribute to shorter operative time. Furthermore, different degrees of postoperative complications occurred in both the ERAS and conventional groups. There were no significant differences in the incidence of cardiovascular diseases, anastomotic leakage, bleeding, chylothorax, incision infection, vocal cord paralysis, and DGE, but the overall incidence of postoperative complications was lower in the ERAS group (26.1% *vs*. 50.4%, P<0.001), especially the incidence of pulmonary complications (5.4% *vs*. 15.1%, P=0.025). Pulmonary complications are the most common adverse events following esophageal surgery and could reach up to 67% of esophageal cancer patients (25). Thus, it is believed that the most significant potential benefit of the ERAS protocol is to reduce the incidence of pulmonary complications.

Pain is an essential factor in postoperative recovery. Pain management remains a core part of ERAS protocol and should be guided from the preoperative period (26). It has been pointed out that effective preoperative pain guidance can reduce postoperative pain, reduce the use of analgesic drugs, and positively affect enhanced recovery (27). In patients with esophageal cancer, postoperative pain mainly comes from injuries caused by endotracheal intubation, surgical procedures, and drainage tubes. Postoperative pain limits the patient’s ability of deep breathing, cough expectoration, and postoperative out-of-bed activities, while increasing the incidence of pulmonary infection (28). A previous study proposed that the decrease of thoracic fluid drainage volume after radical resection of esophageal carcinoma under the concept of ERAS could significantly reduce postoperative pain, promote early ambulation, improve rehabilitation training, and achieve safe and effective rehabilitation (29). Similarly, in our study, postoperative pain scores was significantly lower in the ERAS group than in the conventional group (P<0.001). Meanwhile, the duration of postoperative analgesic pump was shorter in the ERAS group than in the conventional group (P<0.001). Therefore, our ERAS concept effectively reduced postoperative pain and enhanced recovery compared to the conventional group. We also strengthened preoperative and postoperative pain management in the ERAS group in terms of reducing the drainage volume, thereby lightening both physical and psychological pain burden of patients, as well as effectively alleviating the postoperative pain of patients.

Early postoperative ambulation is an essential component of ERAS protocol which can reduce the probability of pulmonary infection and postoperative first flatus time, accelerate the recovery of gastrointestinal function, and facilitate recovery of patients (30, 31). This study indicated that the start time of postoperative ambulation in the ERAS group was significantly shorter than that in the conventional group (P=0.045), and the postoperative first flatus time was also shorter (P<0.001). Besides, the ERAS group had both significantly shorter postoperative hospital stay (P=0.018) and ICU length of stay (P<0.001) than the conventional group. The above results revealed that our ERAS concept allowed patients undergoing RAME to have better out-of-bed activities, accelerate the recovery of intestinal function, shorten the length of hospital stay, and accelerate rehabilitation after surgery. In addition, there remains controversial to provide an early liquid diet after surgery in clinical practice because some scholars worry about the occurrence of anastomotic leakage, but allowing patients to take food early is an essential part of ERAS protocol. For the traditional management mode of MIE, upper gastrointestinal radiography needs to be reexamined about one week after operation to observe the healing of anastomotic stoma, and the patient can gradually have oral feeding without anastomotic leakage (32). Of note, early oral intake has been shown to have positive outcomes with earlier discharge and fewer complications in patients who have undergone upper gastrointestinal resections. However, there is a risk of regurgitation and aspiration pneumonia, and no separate analysis was provided for esophageal anastomoses, which present unique challenges (33). Consequently, our ERAS protocol encouraged patients to perform upper gastrointestinal radiography on POD 5 to ascertain anastomotic integrity and continue a liquid diet. The results of this study showed that the time to liquid diet in the ERAS group was significantly shorter than that in the conventional group (P<0.001). At the same time, there was no significant difference in the incidence of anastomotic leakage (P=0.590). Therefore, we believed that no inevitable relationship existed between anastomotic leakage and early oral feeding in patients after surgery, and an early postoperative liquid diet was safe and feasible.

To our knowledge, this is the first cohort study of ERAS protocol in RAME for esophageal cancer. However, there are some limitations in this study. First, the single-center and retrospective design might limit the externality and generalization of our results. Second, there might exist bias with the retrospective evaluations in terms of the postoperative management strategy selection. Third, the small patient population and lack of blinding may weaken the quality of evidence. More scientific evidence on the ERAS protocol for RAME is required. The lengthy study period including different surgeons may have also affected the study’s findings. However, all surgeons are very skilled in esophagus and upper gastrointestinal surgery. Aside from the restrictions mentioned, specific significant postoperative outcomes, such as first flatus, out-of-bed activity, time to oral feeding, and duration of analgesic pump, were part of the ERAS procedure. Therefore, the ERAS society recommendations need further evaluation and research to unify protocols worldwide.

## 5 Conclusions

In summary, the short-term efficacy of ERAS protocol was comparable, safe, and feasible to those of esophageal cancer treated with RAME, and ERAS protocol showed better postoperative recovery than the conventional treatment in terms of postoperative hospital stay and complications. ERAS protocol could therefore be a superior option for most patients with esophageal cancer. Further randomized trials are needed to verify the advantages of ERAS protocol.

## Data availability statement

The datasets presented in this article are not readily available because to protect the patients’ privacy, the authors declare that raw data collected in this study would remain confidential and would not be available to the public. Requests to access the datasets should be directed to linyidan.academy@foxmail.com.

## Ethics statement

The studies involving human participants were reviewed and approved by Biomedical Ethics Review Committee of West China Hospital of Sichuan University and Jiujiang First People's Hospital. The patients/participants provided their written informed consent to participate in this study.

## Author contributions

XX and JX: Conceptualization, Methodology, Software, Formal analysis, Investigation, Resources, Data curation, Writing – original draft. ZX: Methodology, Software, Formal analysis, Investigation, Resources, Data curation. ZH: Validation, Data curation, Supervision. GA: Validation, Data curation, Supervision. LY: Supervision, Project administration. SX: Supervision, Project administration. YL: Conceptualization, Validation, Writing – original draft, Writing – review and editing, Supervision, Project administration. All authors contributed to the article and approved the submitted version.
